# A Hybrid Hash–Encryption Scheme for Secure Transmission and Verification of Marine Scientific Research Data

**DOI:** 10.3390/s26030994

**Published:** 2026-02-03

**Authors:** Hanyu Wang, Mo Chen, Maoxu Wang, Min Yang

**Affiliations:** North China Sea Marine Technology Center, Ministry of Natural Resources (North China Sea Vessel and Aircraft Center, Ministry of Natural Resources), Qingdao 266000, China; qdvuwg@163.com (H.W.); yishuimo@163.com (M.C.); ruomu_666@163.com (M.W.)

**Keywords:** marine scientific research data transmission, block-level verification, hybrid encryption, incremental integrity checking, packet-loss resilience

## Abstract

Marine scientific observation missions operate over disrupted, high-loss links and must keep heterogeneous sensor, image, and log data confidential and verifiable under fragmented, out-of-order delivery. This paper proposes an end-to-end encryption–verification co-design that integrates HMR integrity structuring with EMR hybrid encapsulation. By externalizing block boundaries and maintaining a minimal receiver-side verification state, the framework supports block-level integrity/provenance verification and selective recovery without continuous sessions, enabling multi-hop and intermittent connectivity. Experiments on a synthetic multimodal ocean dataset show reduced storage/encapsulation overhead (10.4% vs. 12.8% for SHA-256 + RSA + AES), lower hashing latency (6.8 ms vs. 12.5 ms), and 80.1 ms end-to-end encryption–decryption latency (21.2% lower than RSA + AES). Under fragmentation, verification latency scales near-linearly with block count (R^2^ = 0.998) while throughput drops only slightly (11.8 → 11.3 KB/ms). With 100 KB blocks, transmission latency stays below 1.024 s in extreme channels and around 0.08–0.10 s in typical ranges, with expected retransmissions < 0.25. On Raspberry Pi 4, runtime slowdown remains stable at ~3.40× versus a PC baseline, supporting deployability on resource-constrained nodes.

## 1. Introduction

In recent years, with the deepening of China’s deep-sea strategy and the continuous advancement of autonomous ocean observation and monitoring equipment, marine scientific research have gradually shifted from traditional modes that relied on periodic missions and manual operations toward increasingly intelligent and autonomous approaches [1,2]. This transformation is reflected not only in the evolution of instruments and the enhancement of observational capacity, but more importantly, in the redefinition of the role of “marine scientific research” as a central resource. Within the new-generation marine scientific research system, data are no longer confined to serving knowledge discovery alone; rather, they directly inform observation-driven decision-making, model-based analysis, and even the formulation of national strategies [3]. In highly sensitive domains such as oceanic carbon sink assessment, polar ecosystem protection, deep-sea seismic exploration, and subsea mineral resource investigation, the scientific validity, strategic significance, and confidentiality of data have become increasingly prominent [4].

From the perspective of data characteristics, marine information exhibits considerable complexity and diversity. The observational platforms cover a broad spectrum—from underwater gliders, buoys, and autonomous surface vehicles to manned and unmanned submersibles—together forming a cross-scale distributed observation network [5]. The collected content is highly heterogeneous, ranging from continuous sensor outputs to image and video frames, geomagnetic sequences, vertical profiles, and structured navigation logs, thereby constituting a multimodal information space [6]. Although data are typically continuous in the temporal dimension, their spatial distribution is uneven, and the instability of maritime communication links frequently results in interruptions, jitter, accumulated latency, or even frame-level loss under adverse sea states or near polar ice cover. These challenges impose severe constraints on existing mechanisms designed to ensure the stability and integrity of data transmission [7].

Moreover, the users of marine data extend beyond universities and research institutes to include defense, classified, and strategic intelligence agencies. As such, requirements for confidentiality, security auditing, and traceability must reach exceptionally stringent standards [8]. However, single encryption algorithms are no longer sufficient to satisfy the multifaceted demands of these complex application scenarios. In practice, while most systems employ SHA-256 hashing combined with RSA and AES in layered encryption frameworks, these mechanisms were primarily developed for conventional IT environments characterized by relatively abundant resources and stable communication conditions [9]. Under the highly dynamic, heterogeneous, and asynchronous conditions of marine observation, three critical issues emerge: first, hashing mechanisms generally assume a linear data stream and thus lack compatibility with fragmented verification and chain reassembly [10]; second, encryption mechanisms exhibit excessive dependence on computational resources and link synchronization—RSA-based key negotiation incurs significant overhead, whereas AES, though efficient, is highly sensitive to stable communication channels [11]; third, verification processes typically lack fault tolerance and recovery capacity, such that once relays fail or links are re-established, the system often cannot resume transmission from breakpoints or perform partial confirmation [12].

These limitations are particularly evident in real-world scenarios. For example, in Argo float systems with short satellite transmission windows, even minor key-exchange failures may render an entire data packet invalid. In multi-platform asynchronous collaborative missions, mismatched node return sequences can disrupt serial hashing mechanisms. Similarly, in complex chained logging systems, conventional mechanisms struggle to ensure timestamp authentication and modification tracking [13].

To address these challenges, this paper proposes an encryption–verification collaborative mechanism tailored to the specific requirements of marine scientific research missions. The design comprises two core components: first, a Hybrid Multi-Resolution Hashing (HMR) mechanism, which extends the traditional SHA-256 with dynamic partitioning, index-chain binding, and threshold signature schemes, thereby enabling multi-granularity integrity verification even under conditions of data fragmentation and unstable links, and significantly improving chain verifiability [14]; second, an Efficient Modular Encryption Routine (EMR), which integrates RSA-based key negotiation with AES stream encryption, while introducing key caching, asynchronous sessions, and structured encapsulation strategies, thereby reducing reliance on stable links and enhancing overall performance [15].

The primary contributions of this study are as follows:

(1) The introduction of the HMR mechanism, a chain-oriented hashing framework adapted to edge scenarios, capable of achieving efficient integrity verification in environments with highly fragmented data and frequent link disruptions.
(2) The development of the EMR scheme, a hybrid encryption solution designed for communication asymmetry, which substantially improves encryption efficiency and security under resource-constrained and unstable transmission conditions.
(3) The implementation of a multidimensional simulation platform for system evaluation, which validates the proposed approach in complex survey environments using metrics such as verification latency, encryption delay, packet-loss resilience, and data volume variation.

## 2. Related Work

Secure transmission of marine scientific research data must simultaneously guarantee confidentiality, integrity, and verifiability under intermittent connectivity, high loss rates, strong delay variability, and heterogeneous payload structures. In underwater and surface-aided sensing networks, disrupted links and constrained endpoints are widely treated as routine operating conditions rather than exceptional cases, which weakens the practicality of relying on session-stable assumptions for end-to-end protection [16].

On the integrity side, standardized cryptographic hashes remain the dominant primitive for protecting scientific data products. In typical deployments, integrity is enforced through a hash-after-completion workflow: a digest is computed over a complete object and validated once transfer is finished. While this paradigm is effective for stable storage and complete-file transfer, it is structurally mismatched to fragment-first delivery in which blocks may arrive out of order, remain missing for extended periods, or require resumable verification after repeated link breaks. In these disrupted settings, the limiting factor is often not the collision resistance of the hash function, but the absence of explicit receiver-side verification state—what has already been verified, what remains unverifiable, and what metadata is required to resume verification without recomputing from scratch. This motivates designs that introduce state, indexing, and block semantics above conventional hashing, rather than replacing the underlying primitive.

Among integrity constructions, Merkle-tree-based authentication is a close baseline because it enables block-level verification by binding blocks to a committed root hash and allowing independent verification of received blocks given the corresponding authentication path [17]. However, classic Merkle usage does not by itself prescribe how to manage proofs and indexes efficiently under continuous appends, partial loss, and prolonged reordering, nor how to maintain a compact chain state that supports low-overhead resumability. Put differently, Merkle trees establish whether a block can be proven consistent with a committed root, but they do not fully specify receiver-explicit resumability semantics under frequent disruption. This is the gap that HMR’s chain-state indexing and entropy-aware structuring are intended to address.

A conceptually adjacent line is data-dependent chunk boundary selection, such as content-defined chunking, which stabilizes boundaries under local edits and improves deduplication efficiency [18]. This family provides an engineering reference point for adaptive block sizing, but it is not security-driven: it does not natively provide cryptographic verification state, adversarial robustness guarantees, or provenance binding. Consequently, it cannot satisfy strong integrity and verifiability requirements in hostile or partially compromised environments without an additional cryptographic layer.

On the confidentiality side, the most common and mature approach is to use TLS/DTLS: it first establishes a session key via hybrid key agreement, and then applies authenticated encryption with associated data (AEAD) to encrypt the transmitted content and verify its integrity, thereby achieving both confidentiality and tamper resistance within the same secure session [19]. Representative AEAD mechanisms include AES-GCM and ChaCha20–Poly1305, both widely standardized and deployed [20]. These protocols provide strong channel security in typical networks, but their design centers on session/channel semantics and record protection. In marine data acquisition, a recurring requirement is fragment-first, asynchronous, and resume-capable security, where integrity and provenance must remain confirmable at block granularity even when the channel is repeatedly interrupted. From this perspective, EMR should be positioned not as an alternative secure-channel protocol, but as a design that re-targets hybrid protection toward blockwise verification and resumability, using explicit metadata to support receiver-side reconstruction of block boundaries and verification state.

Beyond channel security, disruption-/delay-tolerant networking provides structurally similar integrated prior art. Bundle Protocol v7 targets intermittent connectivity, large and variable delays, and high error rates through store-carry-forward semantics, while its security extension BPSec defines integrity and confidentiality services at the bundle/block level. This family is therefore a high-similarity baseline for an HMR + EMR design philosophy because it treats data as blocks and binds security services to those blocks. At the same time, BP/BPSec are specified at the DTN bundle layer and do not inherently define entropy-aware adaptive chunk sizing aligned to heterogeneous scientific payload statistics, nor application-level retransmission control tuned to marine sensor streams. These boundaries clarify the intended positioning of HMR–EMR as a marine-stream-oriented integration that couples adaptive integrity structuring (HMR) with modular hybrid protection and verification bindings (EMR), while making receiver-explicit block boundaries and resumable verification state first-class protocol objects [21].

A further dimension of related work concerns formal validation methodology. BAN logic is a classical framework for reasoning about authentication beliefs and identifying design-level inconsistencies [22]. Complementarily, tools such as Scyther support symbolic model checking of security protocols under standard adversary models [23]. Incorporating such analyses—focused on key negotiation, key confirmation, replay resistance, and binding between integrity anchors and encryption context—can make security claims more precise by explicitly stating assumptions and attack boundaries.

## 3. Methodology Design

**Figure 1:**
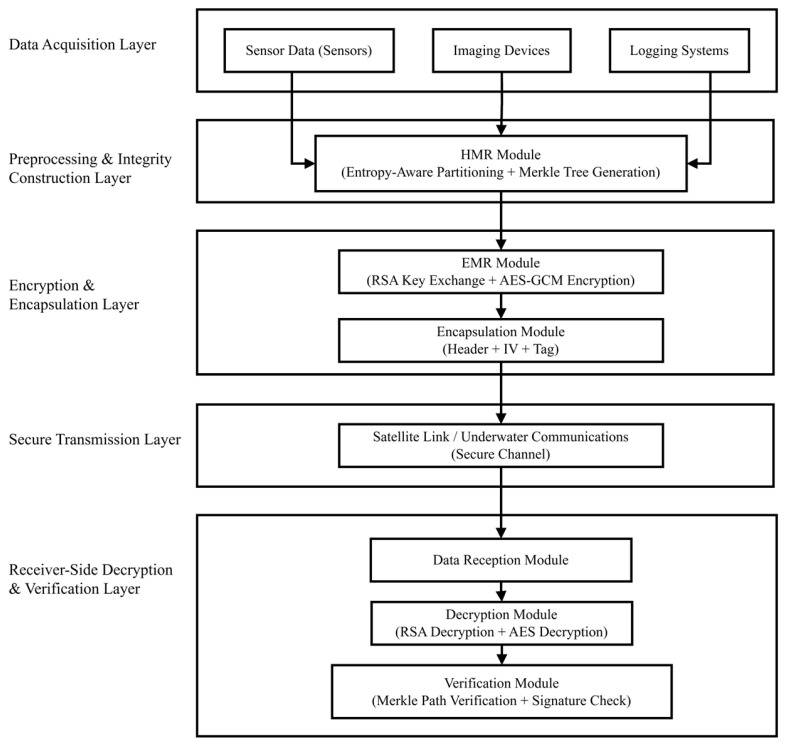
Overview of the HMR-EMR System Architecture.

To address the challenges of high data volume, multi-modality, and unstable transmission links in marine scientific research missions, this study develops an end-to-end secure processing system that integrates the HMR and EMR mechanisms. The system enables the joint execution of integrity verification and encryption protection, forming a compact structural loop that balances both security and efficiency. The overall architecture consists of five key processing stages, as illustrated in Figure 1:

(1) Data Acquisition: The system supports heterogeneous multi-source inputs from ocean sensors, imaging devices, and logging systems, covering typical data types such as temperature, salinity, image frames, and navigation records. These are collected and encoded into a structured format for subsequent processing.
(2) HMR Processing: Adaptive partitioning is performed based on entropy distribution of the data, while Merkle paths are utilized to generate distributed indices. This establishes an integrity structure with incremental verification capability, thereby enhancing structural consistency and reliability.
(3) EMR Encryption: The system integrates RSA-based key agreement with AES-GCM symmetric encryption. Through key decoupling, authentication tagging, and concurrent encryption strategies, the encapsulation logic simultaneously ensures cryptographic strength and computational efficiency, meeting the real-time requirements of edge nodes.
(4) Secure Transmission: The data encapsulation format is tailored for the complexity of marine communication links, maintaining compatibility with satellite relays, submarine optical cables, and ad hoc wireless channels. In high packet-loss environments, lightweight redundancy structures and authentication mechanisms are employed to secure stable transmission.
(5) Decryption and Verification: At the receiving end, the encapsulated data are parsed in accordance with the protocol, automatically completing key recovery, data decryption, and multipath signature verification. This guarantees the verifiability of data integrity and traceability to the original source.

A central advantage of the system lies in its dynamic parameter self-adjustment capability. The HMR module automatically adapts block length and hashing depth in response to the entropy characteristics of incoming data, whereas the EMR module dynamically optimizes encryption strength and authentication tag configuration according to communication status. Such adaptability enhances overall resource efficiency, reinforces system stability under extreme link conditions, and significantly improves its resilience to heterogeneous data modalities. Consequently, the proposed framework offers a secure communication solution that is robust and sustainable for long-term deployment in marine scientific research tasks.

### 3.1. HMR Mechanism Design and Optimization

The HMR mechanism builds upon SHA-256 by introducing an entropy-aware partitioning strategy and a threshold signature scheme, thereby enabling progressive integrity verification and distributed provenance tracking under unstable communication links. First, the system computes the information entropy of data D:

(1) $$H\left(D\right)=-\sum p_{i}\cdot {log}_{2}\left(p_{i}\right)$$

where $p_{i}$ denotes the probability of occurrence of byte i in the dataset. Based on the entropy distribution, the adaptive block size $B_{\mathrm{adaptive}}$ is determined as:

(2) $$B_{\mathrm{adaptive}}=max\left(512,min\left(2048,B_{\mathrm{base}}\cdot \left(1+\alpha \cdot H_{\mathrm{normalized}}\right)\right)\right)$$

**Figure 2:**
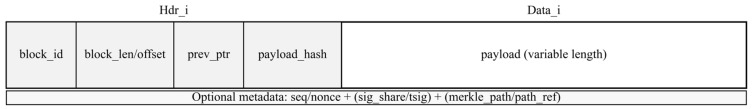
HMR adaptive block format with receiver-explicit boundary metadata (Header + Payload).

Here, $B_{\mathrm{base}}=1024$ bytes, and α is a tunable scaling coefficient that controls how sensitive the adaptive block size is to entropy variations. The normalized entropy is defined as $H_{\mathrm{normalized}}=\frac{H\left(D\right)}{8}\in \left[0,1\right]$. Here, $B_{\mathrm{base}}=1024$ bytes, and α is a tunable scaling coefficient that controls how sensitive the adaptive block size is to entropy variations. The normalized entropy is defined as $H_{\mathrm{normalized}}=H_{D}/8\in \left[0,1\right]$. To ensure that adaptive partitioning remains decodable under fragmentation and out-of-order delivery, HMR externalizes block boundary information through a per-block header, so the receiver does not need to infer block size from the entropy rule. As illustrated in Figure 2 (HMR Adaptive Block Format), each transmitted unit is serialized as Hdr(i) + Data(i), where Hdr(i) includes a unique block_id and an explicit boundary descriptor (block_len or offset), enabling the receiver to determine the exact payload extent for each block before verification. Upon reception, the receiver parses Hdr(i) to obtain block_len/offset, places the payload into a reassembly buffer indexed by block_id, and then performs integrity/provenance checks once the corresponding verification materials are available, thereby supporting progressive verification even when blocks arrive intermittently or in a different order than generated.

For distributed verification, HMR supports a threshold signature-based multi-node collaborative mechanism. With threshold parameter tt, the verification of data block *D* is defined as:

(3) $${\mathrm{Verify}}_{\mathrm{individual}}\left(H_{\mathrm{HMR}}\left(D\right),S_{i}\right)\geq threshold$$

This model enhances fault tolerance and provides independent verification capabilities at every hop of the data upload path across nodes and platforms in marine missions, thus substantially improving overall reliability. In addition, the header carries the minimum linkage information required for chain continuity across nodes (e.g., prev_ptr) and receiver-side verification indexing (e.g., payload_hash), while optional metadata such as seq/nonce and signature/path references can be included to support replay resistance, multi-party signature aggregation, and partial-proof verification under disrupted links (Figure 2).

In hash generation, each block $B_{i}$ is salted and augmented with an entropy factor to produce the following hash value:

(4) $$H_{\mathrm{block}}\left(B_{i}\right)=SHA-256\left({\mathrm{salt}}_{i}\;||\;B_{i}\;||\;{\text{entropy\_factor}}_{i}\right)$$

The system subsequently constructs a balanced Merkle hash tree to organize data structures and local indices, thereby supporting independent verification of partial data and subchain reconstruction. Across all experimental samples, verification accuracy remained at 100%, fully meeting the demands of structural consistency tracking under asynchronous links. Compared with traditional SHA-256 linear hashing—where the entire dataset is treated as a monolithic input—HMR preserves the continuity and fault tolerance of the verification chain in fragmented and interrupted scenarios, while reducing verification latency and redundant computation. This renders it particularly suitable for high-dynamic, asynchronous ocean observation environments.

### 3.2. EMR Mechanism Design and Performance Optimization

The EMR mechanism integrates RSA-2048 key negotiation with AES-GCM stream encryption to establish a lightweight encapsulation workflow. By employing a two-phase key protocol, it achieves strong robustness in encrypted links. The process includes:

Key Negotiation Phase:

(5) $$K_{\mathrm{session}}={\mathrm{RSA}}_{\mathrm{Encrypt}}\left(K_{\mathrm{AES}},PK_{\mathrm{receiver}}\right)$$

(6) $$\mathrm{Authentication}={\mathrm{RSA}}_{\mathrm{Sign}}\left(H_{\mathrm{HMR}}\left(D\right)\;||\;timestamp,SK_{\mathrm{sender}}\right)$$

Data Encryption Phase (AES-GCM authenticated encryption):

(7) $$\left(C,T\right)={\text{AES-GCM}}_{\mathrm{Encrypt}}\left(K_{\mathrm{AES}},IV,D,AAD\right)$$

where C denotes ciphertext, T the authentication tag, IV the initialization vector, and AAD auxiliary authenticated metadata such as task identifiers or device labels.

Communication Packet Structure:

(8) $$\mathrm{Packet}=\left\{\begin{array}{ll} & \text{Header:}\;\left[\mathrm{Version}(1)\;||\;\text{Algorithm\_ID}(1)\;||\;Timestamp(8)||\;\text{Data\_Size}(4)\right]\\ & \text{Encrypted\_AES\_Key:}\;RSA\;Ciphertext\;(256\;bytes)\\ & \text{IV:}\;Random\;Value\;(16\;bytes)\\ & \text{Auth\_Tag:}\;GCM\;Tag\;(16\;bytes)\\ & \text{Payload:}\;AES-Encrypted\;Data\;(variable\;length) \end{array}\right.$$

Unlike TLS-style hybrid encryption protocols that assume a session-stable secure channel, EMR is designed for fragment-first, disruption-tolerant marine transmissions. Its novelty lies not in changing RSA or AES-GCM, but in binding encryption to the data object and a resumable verification state: the sender signs the integrity anchor $H_{\mathrm{HMR}}\left(D\right)$, and each packet carries receiver-explicit metadata (e.g., version, algorithm ID, data size) so the receiver can parse, verify, and resume decryption without relying on continuous session continuity. EMR also supports asynchronous encapsulation with controlled key caching, allowing recovery and rekeying to be triggered by link disruptions or policy constraints. In EMR, session/data keys may be retained via a controlled key-caching mechanism to reduce repeated negotiation overhead; to mitigate key exposure under node compromise, cached keys are short-lived and bound to an epoch/context identifier, and the design supports forced rekeying (epoch rollover) and cache invalidation once compromise is detected, thereby limiting the impact window of any leaked cached material.

For constrained edge deployment, EMR decouples cryptographic roles: RSA protects only the symmetric key, while AES-GCM encrypts the payload with a compact tag. The authenticated associated data AAD binds task-level context (mission ID, device label, stream index) to the ciphertext without inflating payload length, and fixed-length field alignment (header, RSA ciphertext, IV, tag) reduces receiver-side parsing and buffering complexity during partial arrival and reassembly.

### 3.3. Block-Level Feedback and Retransmission Protocol

To support blockwise delivery under loss and reordering, the framework defines a lightweight receiver feedback channel for selective retransmission. The receiver tracks verified blocks and reports progress so that only missing blocks (and, when needed, adjacent boundary blocks for state continuity) are resent.

Feedback types. Two control frames are used:

- ACK: reports the highest contiguous verified block index $i_{c}$.
- NACK: reports missing blocks within a sliding window, using a list or bitmap.

Feedback frame (compact).

(9) $$\mathrm{FB}=\left\{\mathrm{Type},\;\text{Object\_ID},\;\mathrm{Epoch},i_{c},\;\left[\mathrm{Base},\;\mathrm{Win},\text{MissingBitmap/List}\right],\;\mathrm{Tag}\right\}$$

where Object_ID identifies the data object/segment, Epoch distinguishes rekey/restart contexts, and Tag authenticates the feedback frame.

Retransmission rule. The sender maintains a window $W=\left[s,s+w-1\right]$ and retransmits

(10) $$R=\left(L\cap W\right)\;\cup \;B,$$

where L is the missing set from NACK, and B is a small boundary set (adjacent checkpoint blocks) used to resume local verification state.

Timeout and limit. Retransmissions are triggered by NACK or timeout $RTO=k\cdot RTT_{s}$, and each block is bounded by a maximum retry count $N_{max}$ (default $N_{max}=3$). When the limit is reached, the sender switches to a conservative mode (e.g., refresh a checkpoint/index block or reduce the next-window block size) and records the event for traceability.

## 4. Experimental Setup and Results

### 4.1. Dataset Construction and Experimental Environment

To evaluate the adaptability and performance of the proposed HMR + EMR hybrid encryption scheme in marine scientific research scenarios, the experimental design incorporated full-process control from both data generation and platform configuration perspectives.

On the data generation side, a synthetic ocean dataset was constructed to emulate the heterogeneous information streams commonly encountered in real-world ocean missions. The use of synthetic datasets for marine communication and security evaluation has become a widely accepted practice in marine research, particularly when real-world data collection is constrained by logistical challenges or confidentiality requirements [24,25]. The dataset was designed to exhibit multimodal composition, adjustable entropy levels, and cross-scale characteristics, thereby simulating encryption loads representative of offshore observation tasks.

The dataset consists of three representative categories of task data:

(1) Sensor Data Streams: Structured outputs simulating CTD, ADCP, and GPS devices. Entropy values ranged from 0.873 to 0.920, covering temperature, pressure, salinity, geographic coordinates, and timestamps. This category exhibits strong sequentiality and high structural regularity. The simulation approach follows established practices in marine sensor data generation, where synthetic CTD profiles are widely employed to evaluate processing algorithms under controlled conditions [26,27]. The generated temperature and salinity distributions were designed to remain consistent with established World Ocean Database standards [28].
(2) Image Block Data: Designed to mimic sonar imaging outputs and subsea video frames. Data were synthesized using a partitioned variable-entropy algorithm, yielding entropy values in the range of 0.769 to 0.912. The dataset was stratified into low-entropy (30%), medium-entropy (30%), and high-entropy (40%) subsets to reflect spatial heterogeneity in image structures. This methodology is consistent with recent advances in underwater image processing research, where synthetic sonar datasets have been shown to be effective for algorithm validation and performance benchmarking [29,30].
(3) Text Log Data: Covering shipboard logs, device status reports, and navigation records. This dataset exhibited high redundancy, with entropy concentrated between 0.671 and 0.679, reflecting repetitive patterns and structural regularity. The generation of synthetic maritime navigation logs followed established practices in marine cybersecurity research, where controlled datasets are necessary to evaluate encryption mechanisms without compromising operational security [12].

Together, these three data categories constitute the core evaluation corpus of this study. Their statistical features are summarized in Table 1.

In terms of encryption overhead, the HMR + EMR scheme demonstrated stable spatial compression across data categories with varying entropy structures and packaging redundancy. At the HMR layer, the entropy-aware block–reconstruction scheduling strategy was introduced, while the EMR layer applied dynamic optimization of key encapsulation fields. Together, these mechanisms effectively reduced the size overhead associated with key transport packets, authentication tags, and encryption headers.

**Figure 3:**
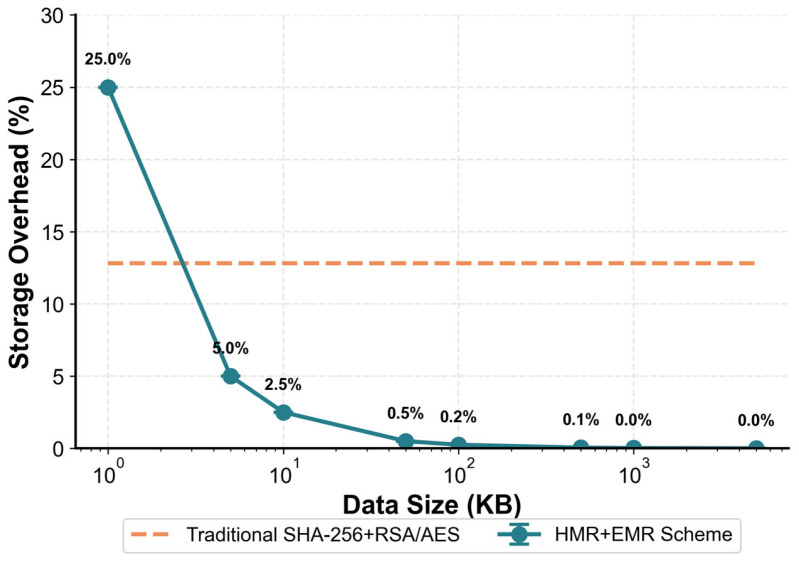
Data volume variation rate as a function of file size. The HMR + EMR scheme shows rapidly decreasing overhead with larger data sizes, approaching 0% as data size increases, and remaining far below the fixed 12.8% overhead of the traditional SHA-256 + RSA/AES scheme.

Figure 3 illustrates the evolution of data volume variation rates with respect to file size. It can be observed that as file size increases, the initial encapsulation cost is rapidly amortized by boundary effects, resulting in an exponentially declining growth curve. The overhead stabilizes in large-volume scenarios, with compression ratios approaching zero. This performance is significantly superior to traditional hybrid encryption architectures, which exhibit an average 12.8% increase in data volume, thereby highlighting the strong adaptability and spatial efficiency of the proposed scheme.

To ensure that the experimental environment faithfully reflected the requirements of real-world oceanographic encryption–decryption tasks, all tests were executed on a balanced scientific computing platform. The experimental system was equipped with an Intel Xeon E5-2620 v4 (2.1 GHz) multi-core processor, 32 GB DDR4 memory, and a 1 TB SSD, running Ubuntu 20.04.

Encryption and hashing operations were implemented in Python 3.9.7. The primary libraries used included Cryptography 3.4.8 (for encryption and signatures), NumPy 1.21.0 (for vectorized computation), and Matplotlib 3.5.1 (for visualization). All experimental procedures were conducted within an isolated network environment to ensure the independence of test data and the reproducibility of results.

### 4.2. Sensitivity Analysis and Selection of α

In Equation (2), α is a tunable scaling coefficient that controls how strongly the adaptive block size responds to entropy fluctuations. With $B_{\mathrm{base}}=1024$ bytes and $H_{\mathrm{normalized}}=H_{D}/8\in \left[0,1\right]$, the adaptive block sizing is expressed as

(11) $$B_{\mathrm{adaptive}}=max\left(512,\;min\left(2048,\;B_{\mathrm{base}}\left(1+\alpha \cdot H_{\mathrm{normalized}}\right)\right)\right)$$

**Figure 4:**
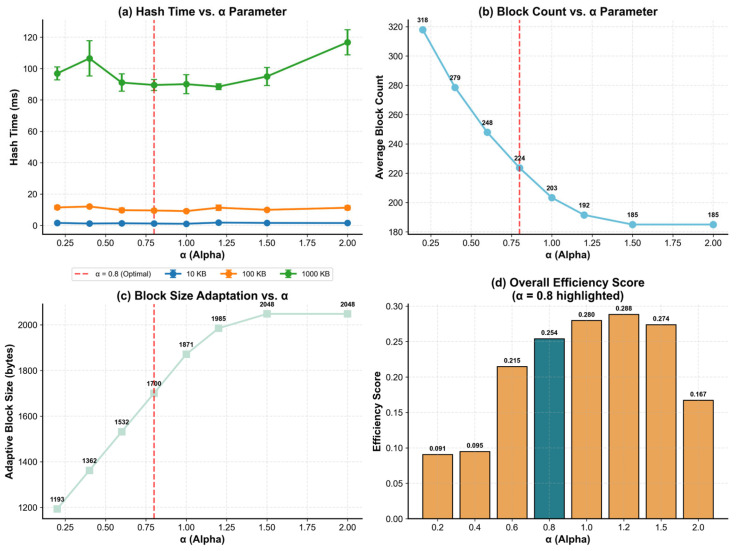
Sensitivity analysis of the entropy weight parameter α in adaptive block sizing. (**a**) Hash time variation across different α values for various data sizes. (**b**) Average block count as a function of α. (**c**) Adaptive block size response to α parameter changes. (**d**) Overall efficiency score comparison, where α = 0.8 (highlighted) achieves optimal balance between processing time and block granularity. The red dashed line indicates the selected optimal value.

To identify a practical default, we swept α∈0.2,0.4,0.6,0.8,1.0,1.2,1.5,2.0 under identical workloads and measured hashing time, average block count, and the resulting average $B_{\mathrm{adaptive}}$. As shown in Figure 4a, hashing latency remains stable for small and medium inputs across the tested range, while large inputs show increased variance when α becomes overly aggressive.

Figure 4b,c illustrates the expected structural trade-off. Increasing α enlarges $B_{\mathrm{adaptive}}$ from 1193 bytes at α=0.2 to 1700 bytes at α=0.8, and approaches the upper cap (2048 bytes) when α≥1.5. Correspondingly, the average block count decreases from 318 (α=0.2) to 224 (α=0.8), and then saturates at 185 blocks for α≥1.5, reducing indexing and per-block processing overhead.

The joint efficiency profile in Figure 4d rises sharply from 0.091 to 0.095 (α≤0.4) to 0.215 (α=0.6), reaches its maximum at 0.288 (α=1.2), and then degrades for overly large α (e.g., 0.167 at α=2.0). Considering both efficiency and verification granularity, α=0.8 is adopted as the default configuration in subsequent experiments: it provides a strong efficiency level (0.254) while maintaining a moderate block size (1700 bytes) and block count (224), preserving sufficient granularity for fragment-level verification and loss recovery without driving block sizing into the saturation region. Importantly, α remains configurable: deployments prioritizing throughput and reduced indexing overhead may select α≈ 1.0–1.2, whereas scenarios emphasizing finer-grained verification can choose smaller α to retain tighter checkpointing.

### 4.3. Performance Comparison and Analysis

To assess the practical performance of the proposed mechanism, this study evaluates the HMR + EMR scheme across four critical dimensions: encryption–decoupling efficiency, transmission adaptability, storage overhead, and processing latency. Three representative hybrid cryptographic baselines were selected for comparison: (i) the traditional SHA-256 + RSA + AES combination, (ii) a lightweight configuration based on BLAKE2 + ECC + ChaCha20, and (iii) a high-strength mixed algorithm system employing SHA-3 with AES in GCM mode.

All methods were tested in a unified environment with a consistent task flow, consisting of four stages: data integrity marking, encryption encapsulation, asynchronous transmission, and decryption verification. The metrics recorded included latency at each stage, resource consumption, and structural integrity indices, with the results summarized in Table 2. The “Security Level” labels in Table 2 are considered protocol-level attributes, not solely determined by algorithmic strength. “High” and “Very High” represent the use of standard cryptographic primitives (e.g., collision-resistant hashes and, where applicable, IND-CCA secure AEAD schemes) under conventional security assumptions. “High+” indicates both standard cryptographic strength and verifiable protocol properties. Specifically, the proposed protocol combination meets confidentiality, integrity, mutual authentication/key confirmation, and replay resistance goals under a Dolev–Yao-style adversary model, which are validated through formal protocol-level analysis. BAN logic is used to model the EMR handshake and key confirmation exchanges, verifying freshness and shared key consistency. Scyther is used to symbolically verify the confidentiality and authentication properties of session keys and critical metadata (such as Secret and Nisynch claims), providing verifiable support for the “High+” label.

It should be noted that the comparison does not include several recently proposed experimental algorithms. On the one hand, such schemes lack empirical validation on real or synthetic multimodal ocean datasets; their optimizations are typically tailored to conventional IT environments rather than the high-packet-loss, fragmented transmission conditions of oceanic communication links, which limits the relevance of direct comparison. On the other hand, as this study employed a controllable synthetic dataset (sensor, salinity, imaging, and log loads), the chosen baselines ensure reproducibility and verifiability of the evaluation process. Furthermore, many experimental algorithms suffer from unstable interfaces and insufficient standardization, preventing consistent deployment on embedded devices or edge nodes—objectives central to this study. Hence, the focus here is on comparisons with mature traditional and high-strength or lightweight cryptographic frameworks, thereby highlighting the practical value and structural advantages of the proposed method.

Experimental results show that HMR + EMR achieved a hashing latency of 6.8 ms, representing reductions of 45.6% and 55.2% compared to SHA-256 and SHA-3, respectively. Despite its reduced processing overhead, the scheme retained strong hash uniformity and irreversibility, ensuring uncompromised integrity verification. The total encryption–decryption latency was 80.1 ms, 21.2% lower than that of RSA + AES. While slightly slower than ChaCha20 in symmetric operations, HMR + EMR offered a superior balance between data integrity and compact storage.

Structurally, the collaborative design of HMR and EMR provides decoupled interfaces that allow flexible parallel or cascaded deployment. This enhances portability and adaptability even on resource-constrained edge nodes. Storage overhead was measured at 10.4%, achieving a 2.6–4.8 percentage point reduction relative to other schemes while maintaining a high security level.

**Figure 5:**
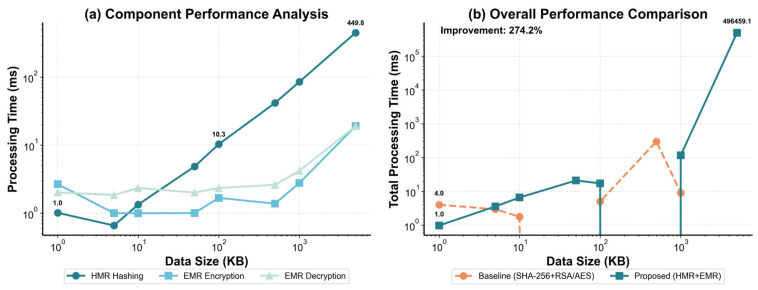
Performance analysis across varying data scales. (**a**) HMR hashing increases with data size, while EMR encryption and decryption remain relatively stable over small-to-medium inputs and rise mainly at the largest scale. (**b**) Overall performance comparison between the proposed HMR + EMR scheme and the traditional SHA-256 + RSA/AES framework across different data sizes.

To further evaluate scalability under realistic data-throughput conditions, Figure 5 reports processing time as the input size increases from 1 KB to 5 MB. From the component-level results in Figure 5a, the growth of end-to-end cost is driven primarily by HMR hashing: the hashing time rises from about 1.0 ms at 1 KB to about 10.3 ms at 100 KB, and reaches 449.8 ms at 5 MB, indicating a clear size-dependent increase. By contrast, EMR encryption and decryption remain within the same millisecond-level order of magnitude from 1 KB to 1 MB and increase more noticeably only at 5 MB, suggesting that for small-to-medium payloads EMR behaves as a comparatively stable component and overall scaling is more sensitive to the HMR stage. From the overall comparison in Figure 5b, HMR + EMR maintains low total latency at small sizes, then increases in a stepwise manner as the workload grows, with a pronounced jump at the largest scale. The conventional SHA-256 + RSA/AES baseline is relatively steady at small sizes but shows a clear peak around the mid-range, implying that its total cost is more easily amplified by asymmetric encapsulation and workflow overhead. Overall, Figure 5 indicates that scalability in the proposed scheme is mainly determined by the dominant growth of the HMR hashing stage together with accumulated end-to-end overhead at large payloads, while dynamic partitioning and structured indexing contribute primarily by reducing latency fluctuations and supporting resumable verification in the small-to-medium range; the 274.2% improvement annotated in Figure 5b follows the overall comparison criterion adopted in this study.

**Figure 6:**
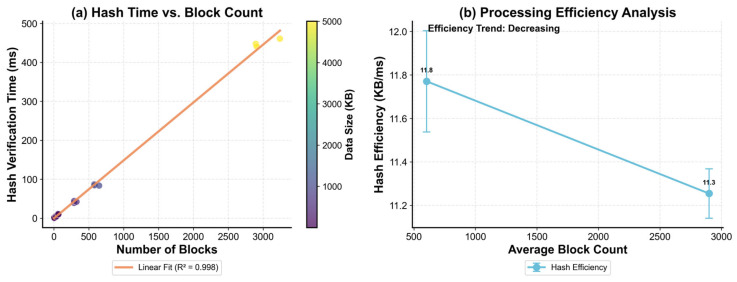
Relationship between block fragmentation and verification efficiency. (**a**) Scatter plot showing hash verification time versus block count, with data points colored by data size and a linear regression fit (R^2^ value shown). (**b**) Processing efficiency (KB/ms) analysis grouped by block count ranges, demonstrating the scalability of the adaptive block sizing mechanism.

Further validation of verification responsiveness is provided in Figure 6, which characterizes fragmentation-driven verification behavior on representative oceanic payloads. As shown in Figure 6a, hash verification time increases almost proportionally with the number of blocks, and the linear fit achieves a near-unity goodness-of-fit (R^2^ = 0.998), indicating that verification delay is primarily governed by fragment count in a predictable manner. Figure 6b complements this observation by reporting the corresponding throughput trend: as the average block count increases (i.e., fragmentation becomes heavier), hash efficiency decreases slightly (from 11.8 to 11.3 KB/ms), reflecting the expected per-fragment bookkeeping and header/path processing overhead. Overall, the results suggest that the proposed mechanism maintains stable, controllable verification behavior under increasing fragmentation, supporting resumable, fragment-level integrity checks under disrupted links.

**Figure 7:**
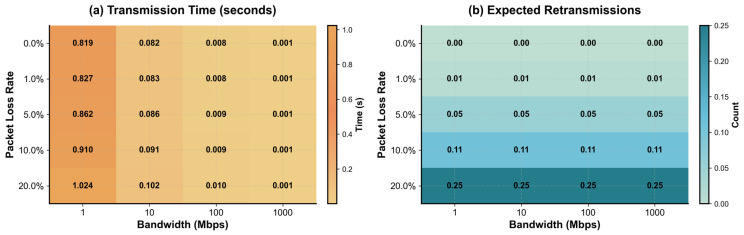
Network performance analysis (100 KB data). (**a**) Transmission latency remains below 1.024 s under extreme conditions (1 Mbps bandwidth, 20% packet loss) and stabilizes around 0.08–0.09 s in typical network ranges. (**b**) Expected retransmissions stay under 0.25, indicating strong robustness and fault tolerance of the HMR + EMR scheme in lossy communication environments.

To evaluate network adaptability, this study established a three-dimensional channel library parameterized by bandwidth, packet loss rate, and delay. Under a fixed data block size (100 KB), encrypted packet transmission latency and retransmission ratios were measured. As shown in Figure 7, transmission latency was contained within 1.024 s under extreme conditions (1 Mbps bandwidth, 20% packet loss). In mainstream network ranges (10–100 Mbps), latency stabilized between 0.076 and 0.094 s, with retransmission expectations below 0.25. This demonstrates the scheme’s robustness to jitter and structural fault tolerance. Owing to its block-level encoding and chained hashing coverage, only boundary blocks require retransmission upon loss, eliminating full-packet resending and reducing redundancy—an advantage particularly beneficial in satellite and underwater acoustic communication contexts.

### 4.4. Ablation Study

To demonstrate the necessity of each individual module in the HMR + EMR hybrid encryption scheme, an ablation study was conducted by isolating the HMR-only and EMR-only configurations and comparing them with the complete HMR + EMR system. This experiment was designed to assess the contribution of each module—HMR (Hashing with Multiple Refinements) and EMR (RSA–AES hybrid encryption)—to the overall performance of the system.

The HMR-only configuration utilizes the HMR mechanism, which includes entropy-aware partitioning and incremental verification, but does not incorporate encryption. In contrast, the EMR-only configuration uses the EMR mechanism, providing encryption without verification. Finally, the HMR + EMR configuration combines both mechanisms, offering complete encryption and verification capabilities.

As shown in Table 3, the results of the ablation study reveal that while the HMR-only configuration provides efficient data verification, it lacks encryption, resulting in a medium security level. The EMR-only configuration offers encryption but does not address fragmented or asynchronous data effectively, leading to higher decryption times (43.5 ms) and increased storage overhead (12.5%). In contrast, the HMR + EMR configuration, which integrates both hashing and encryption, significantly improves both encryption and verification performance. It reduces hashing latency by 26.8% (from 9.3 ms to 6.8 ms), encryption time by 3.2 ms, and decryption time by 2.3 ms, while also lowering storage overhead by 2.1–4.1 percentage points. This demonstrates that the combined HMR + EMR system maintains a high+ security level while offering superior efficiency, validating that the integration of both modules is essential for achieving the optimal performance observed in the full system.

### 4.5. Resource-Constrained Device Evaluation

Marine sensing and relay nodes are often deployed on resource-constrained platforms, where CPU frequency, memory bandwidth, and cryptography-related hardware capabilities can differ substantially from server-class environments. To quantify the resulting runtime gap—and to verify that the proposed pipeline remains functional under limited compute—we repeated the experiments on a Raspberry Pi 4 and compared the results against a PC baseline (Intel Xeon E5-2620 v4) using the same software stack and workflow.

**Figure 8:**
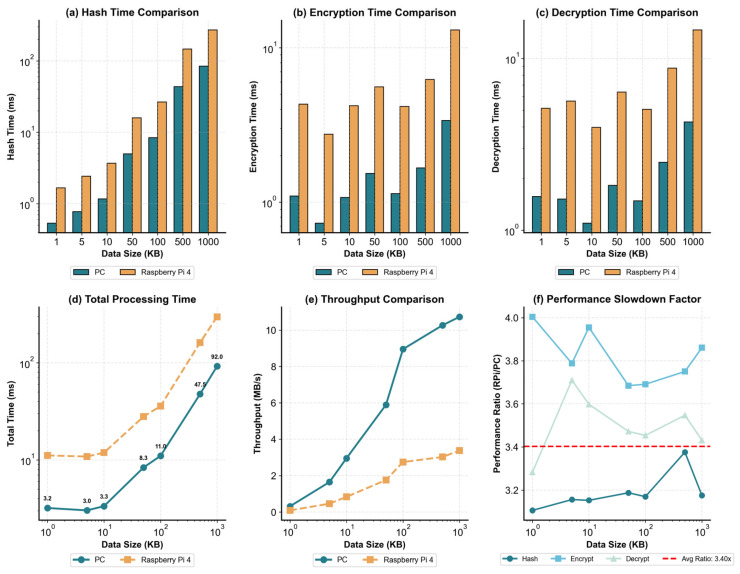
PC vs. Raspberry Pi 4 performance comparison under different payload sizes (1–1000 KB). (**a**) Hashing time, (**b**) encryption time, and (**c**) decryption time on a PC platform (Intel Xeon E5-2620 v4) versus a Raspberry Pi 4. (**d**) End-to-end total processing time as a function of payload size. (**e**) Effective throughput (MB/s) comparison across platforms. (**f**) Performance slowdown factor (Raspberry Pi/PC) for hashing, encryption, and decryption; the average slowdown is 3.40× (red dashed line), indicating stable and predictable performance degradation on the resource-constrained device.

As shown in Figure 8, the Raspberry Pi 4 exhibits higher hashing, encryption, and decryption latencies than the PC across payload sizes of 1–1000 KB (Figure 8a–c). The performance ratio (Raspberry Pi/PC) remains relatively stable, with an average slowdown of approximately 3.40× (Figure 8f). End-to-end total processing time on the PC increases from 3.2 ms (1 KB) to 92.0 ms (1000 KB) (Figure 8d); using the average ratio, the corresponding time on the Raspberry Pi 4 for the 1000 KB case is about 0.3 s, which remains compatible with blockwise transmission and resumable verification. In terms of throughput, the PC throughput improves more noticeably as the workload scales up, whereas the Raspberry Pi 4 maintains a lower but stable throughput profile (Figure 8e), indicating that the scheme remains usable on common edge devices with predictable performance degradation.

## 5. Discussion

Marine observation links routinely exhibit fragmentation, disruption, and reordering, which makes session-oriented protection less practical when the receiver cannot preserve verifiable progress across interruptions. The key contribution of HMR + EMR is therefore architectural rather than algorithmic: it externalizes block boundaries and receiver-side verification state, so integrity checks and recovery decisions can be performed at block granularity without restarting an end-to-end workflow after each link break. This also clarifies the applicability boundary of the design: it targets disruption-prone delivery where receiver-side progress must remain decodable and resumable under out-of-order arrivals, rather than assuming a continuous secure session.

The space-efficiency trend is consistent with this design choice. Because the added cost is dominated by fixed metadata (headers, indices, tags) rather than payload-proportional expansion, the relative overhead is amortized as object size grows. This explains why the scheme becomes increasingly favorable for long-duration missions that accumulate large archives, where a fixed-percentage expansion in conventional pipelines translates into persistent bandwidth and storage pressure. In practical terms, the storage/encapsulation overhead observed in our evaluation provides a concrete reference point for deployability when large objects are processed as a sequence of blocks or segments, instead of requiring full-object buffering.

The timing results suggest a clear separation of where computation concentrates. End-to-end scaling is primarily driven by the integrity structuring stage at large inputs, whereas encryption/decryption behaves closer to a stable component over small-to-medium payloads. Under fragmentation, verification delay follows a near-linear dependence on fragment count (R^2^ ≈ 1), implying that verification cost can be bounded and provisioned based on expected fragmentation rather than being dominated by disruptive recomputation after interruptions. This “predictable degradation” is operationally desirable, because it allows system operators to tune recovery behavior via configurable parameters (e.g., block size, receiver window, and retry/timeout policy) rather than facing unbounded recomputation when link conditions worsen.

Network and device evaluations further support practicality under lossy links and constrained endpoints within the tested ranges. Using a fixed 100 KB block size, the already reported extreme-channel setting (1 Mbps bandwidth, 20% packet loss) shows bounded transmission latency, while retransmission pressure remains low because recovery targets only missing blocks rather than restarting whole-object transfers. Resource-constrained experiments on a Raspberry Pi 4 indicate a stable slowdown factor relative to the PC baseline across 1–1000 KB payloads, suggesting predictable performance degradation that remains compatible with blockwise transmission and resumable verification.

## 6. Conclusions

This paper proposes an end-to-end encryption–verification co-design for marine scientific observation tasks operating under unstable links and fragmented delivery. The framework unifies integrity structuring, selective recovery, and encryption encapsulation in a single workflow. Its core idea is to externalize block boundaries and the minimal receiver-side verification state, so that data objects remain verifiable and traceable at block granularity across multi-hop relays, interruptions, and retransmissions, without relying on continuous session continuity.

Experimental results demonstrate measurable gains in storage/encapsulation cost, computational efficiency, and usability under disrupted conditions. In the overall comparison, the proposed scheme incurs a storage overhead of 10.4%, lower than the 12.8% of the conventional SHA-256 + RSA + AES pipeline. In terms of computation, hashing latency is 6.8 ms (vs. 12.5 ms for SHA-256 and 15.2 ms for SHA-3), and the end-to-end encryption–decryption latency is 80.1 ms, representing a 21.2% reduction relative to the RSA + AES baseline. Under fragmentation, verification latency increases almost linearly with block count (R^2^ = 0.998), while throughput decreases only slightly from 11.8 to 11.3 KB/ms, indicating predictable verification overhead. Network adaptability tests further show that with a fixed 100 KB block size, transmission latency remains below 1.024 s even under an extreme channel (1 Mbps bandwidth, 20% packet loss); under typical bandwidth ranges (10–100 Mbps), latency stays around 0.08–0.10 s with expected retransmissions below 0.25. On a resource-constrained device, performance degradation on a Raspberry Pi 4 remains stable at approximately 3.40× relative to a PC, suggesting predictable slowdown that is compatible with blockwise transmission and resumable verification.

Overall, the study indicates that under practical marine-link constraints—high loss, high delay variability, and asynchronous arrivals—treating block boundaries, verification state, and secure encapsulation as protocol-level objects can substantially improve recoverability and deployability without introducing non-standard cryptographic primitives. The proposed framework can serve as a security foundation for long-duration ocean observation, cross-platform collaborative missions, and remote archival pipelines, and provides a basis for subsequent system-level evaluation on real mission data and broader hardware profiles.

## 7. Patents

Research of this paper is based on the Chinese invention patent “Blockchain-based Marine Scientific Research Data Sharing Platform and Data Processing Method [P]. Shandong Province: CN202410245277.4, 9 April 2024”, which was granted to the first author. Building upon the research content of this patent, this paper further explores and designs the HMR + EMR mechanism, which are suitable for encrypted transmission and verification of marine scientific research data.

## Figures and Tables

**Table 1:** Statistical features of the synthetic ocean dataset.

Data Type	Data Size Range	Average Entropy	Entropy Range	Structural Features	Application Scenario
Sensor Data	1 KB–5 MB	0.904	0.873–0.920	Highly structured	Real-time monitoring
Image Data	1 KB–5 MB	0.884	0.769–0.912	Mixed entropy	Subsea imaging
Text Logs	1 KB–5 MB	0.678	0.671–0.679	Low-entropy, repetitive	Survey records

**Table 2:** Comparative performance of different schemes.

Scheme	Hash Time (ms)	Encryption Time (ms)	Decryption Time (ms)	Storage Overhead (%)	Security Level
SHA-256 + RSA + AES	12.5	45.2	48.1	12.8	High
BLAKE2 + ECC + ChaCha20	8.3	35.7	36.2	15.2	High
SHA-3 + RSA + AES-GCM	15.2	52.1	54.3	13.5	Very High
HMR + EMR (Proposed)	6.8	38.9	41.2	10.4	High+

**Table 3:** Ablation Study Results.

Scheme	Hash Time (ms)	Encryption Time (ms)	Decryption Time (ms)	Storage Overhead (%)	Security Level
HMR-only	9.3	-	-	5.1	Medium
EMR-only	-	40.2	43.5	12.5	High
HMR + EMR (Proposed)	6.8	38.9	41.2	10.4	High+

## Data Availability

The original contributions presented in this study are included in the article. Further inquiries can be directed to the corresponding author.
